# Overexpression of p53 protein in primary Ewing's sarcoma of bone: relationship to tumour stage, response and prognosis

**DOI:** 10.1038/sj.bjc.6690190

**Published:** 1999-03

**Authors:** A Abudu, D C Mangham, G M Reynolds, P B Pynsent, R M Tillman, S R Carter, R J Grimer

**Affiliations:** Royal Orthopaedic Hospital Oncology Service, Birmingham, B31 2AP, UK

**Keywords:** p53, Ewing's sarcoma, tumour suppressor

## Abstract

Biopsy tissues of 52 patients with Ewing's sarcoma of bone treated between 1983 and 1993 were examined immunohistochemically to determine the significance of p53 protein in diagnosis and prognosis of Ewing's sarcoma. Mean age at diagnosis was 17 years (range 6–36) and minimum follow-up was 30 months. The tumours were located in the extremities and central bones in 35 and 17 patients respectively. Metastases were present in seven patients at diagnosis. Treatment consisted of chemotherapy, surgery and/or radiotherapy in all the patients. Overexpression of p53 protein was demonstrated in seven patients (14%). There was no relationship between expression of p53 and site of tumours. Patients who overexpressed p53 protein appeared to have more advanced diseases at diagnosis and poorer response to chemotherapy than those without p53 overexpression. The 5-year relapse-free survival and overall survival in patients without metastases at the time of diagnosis were 66% and 71%, respectively, in p53 protein-negative patients compared with 20% relapse-free and overall survival in those with p53 protein overexpression (*P* = 0.01). The poorer prognosis in p53 protein-positive patients was independent of site, local treatment or necrosis of the tumours (*P* < 0.05). Over-expression of p53 protein is an independent poor prognostic factor in Ewing's sarcoma of bone. © 1999 Cancer Research Campaign


*British Journal of Cancer* (1999) **79**(7/8), 1185–1189
© 1999 Cancer Research Campaign
Article no. bjoc.1998.0180

### Overexpression of p53 protein in primary Ewing's sarcoma of bone: relationship to tumour stage, response and prognosis

**A Abudu, DC Mangham, GM Reynolds, PB Pynsent, RM Tillman, SR Carter and RJ Grimer**

Royal Orthopaedic Hospital Oncology Service, Birmingham B31 2AP, UK

**Summary** Biopsy tissues of 52 patients with Ewing's sarcoma of bone treated between 1983 and 1993 were examined immunohistochemically to determine the significance of p53 protein in diagnosis and prognosis of Ewing's sarcoma. Mean age at diagnosis was 17 years (range 6–36) and minimum follow-up was 30 months. The tumours were located in the extremities and central bones in 35 and 17 patients respectively. Metastases were present in seven patients at diagnosis. Treatment consisted of chemotherapy, surgery and/or radiotherapy in all the patients. Overexpression of p53 protein was demonstrated in seven patients (14%). There was no relationship between expression of p53 and site of tumours. Patients who overexpressed p53 protein appeared to have more advanced diseases at diagnosis and poorer response to chemotherapy than those without p53 overexpression. The 5-year relapse-free survival and overall survival in patients without metastases at the time of diagnosis were 66% and 71%, respectively, in p53 protein-negative patients compared with 20% relapse-free and overall survival in those with p53 protein overexpression ($P = 0.01$). The poorer prognosis in p53 protein-positive patients was independent of site, local treatment or necrosis of the tumours ($P < 0.05$). Over-expression of p53 protein is an independent poor prognostic factor in Ewing's sarcoma of bone.

**Keywords:** p53; Ewing's sarcoma; tumour suppressor

Ewing's sarcoma is a rare form of primitive, highly cellular malignant round-cell tumour of bone and soft tissue originally described by Ewing (1921). It is the second most common primary malignant tumour of bone diagnosed in childhood after osteosarcoma (Glass and Fraumeni, 1970), and accounts for about 10% of all primary malignant bone tumours (Larsson and Lorentzon, 1974; Price and Jeffree, 1977). The important prognostic factors at the time of diagnosis include the presence or absence of metastases (Cangir et al, 1990; Huvos, 1991) and, possibly, the volume of tumour at diagnosis (Mendenhall et al, 1983; Sauer et al, 1987). There is currently no histological grading system to indicate biological aggression of the tumour in patients with localized Ewing's sarcoma at diagnosis because of the highly primitive nature of this tumour. Identification of factors which may indicate the biological aggression of tumours in different patients with primary Ewing's sarcoma of bone will be beneficial for the treatment of patients with localized disease at the time of diagnosis.

The wild-type *p53* tumour-suppressor gene is located on the short arm of chromosome 17 in the region of 17p13 and encodes for a 53-kDa nuclear phosphoprotein with sequence-specific DNA-binding properties. The *p53* gene product is a transcription factor which has two main functions: (i) it acts to maintain genetic stability and induce cell cycle arrest by inhibiting cells from entering the S-phase of the cell cycle and (ii) to induce apoptosis in the presence of genotoxicity (Lane, 1993). The wild-type p53 protein has a short half-life of 20–30 min in most cells and is considered to be undetectable because of its rapid elimination (Levine, 1992).

Mutation of the *p53* gene is the most common genetic abnormality associated with malignancy (Greenblatt et al, 1994). The product of mutant *p53* gene is characterized by conformational change of the protein with resultant prolonged half-life and stability and a loss of the ability to arrest cell cycle or induce apoptosis. It may further inhibit the activity of the wild-type *p53* protein. Numerous studies have shown a close positive correlation between mutations of *p53* gene and overexpression of p53 protein (Bartek et al, 1990; Davidoff et al, 1991; Esrig et al, 1993). This close correlation is maintained in paraffin-embedded formalin-fixed archived tissues with most antibodies giving similar results (Bass et al, 1994), but it is unclear whether any differences exist between antigen retrieval in archived pufffin-embedded formalin-fixed tissues and fresh tumour tissues.

The exact role of the *p53* tumour-suppressor gene in the primary development of malignancies is still unclear. Mutations of the *p53* gene have been implicated in a variety of carcinomas and sarcomas, and there is increasing evidence in the literature that these abnormalities are associated with high histological grade and adverse prognosis (Isola et al, 1992; Hsu et al, 1993; Toguchida et al, 1993; Cordon-Cardo et al, 1994; Mangham et al, 1995). We have used immunohistochemisty to investigate abnormalities of *p53* gene protein in patients with primary Ewing's sarcoma with a

*Received 20 January 1997*
*Revised 14 July 1998*
*Accepted 3 August 1998*

*Correspondence to:* A Abudu, Royal Orthopaedic Hospital Oncology Service, Northfield, Birmingham B31 2AP, UK

view to determining its significance in the diagnosis and prognosis of this disease.

### MATERIALS AND METHODS

Immunohistochemistry for p53 protein was performed on 4-μm sections of archived formalin-fixed and paraffin-embedded pretreatment biopsy specimens of 52 patients (63%) out of a total of 83 patients, who had diagnostic biopsy and treatment of primary Ewing's sarcoma of bone at our centre between 1983 and 1993. Thirty-one patients were excluded from the study because the original biopsy tissues could not be traced in 18 patients, sufficient tissues were not available for examination in 11, and two were reclassified as small-cell osteosarcoma (one) and chronic osteomyelitis (one).

All the tumours studied were composed of sheets of small round cells with pale, poorly delineated cytoplasm rich in glycogen and with single monotonous round to oval nuclei with frequent mitosis and inconspicuous nucleoli. Variable amounts of necrosis and haemorrhage were often present. There was negative immunostaining for leucocyte common antigen (CD45), desmin, S-100 and cytokeratin. There was positive staining for vimentin and MIC-2GP (CD99).

The avidin–biotin complex method for immunostaining was used for each section, with monoclonal antibody PAb 1801 (Novocastra, UK) at a predetermined optimal dilution of 1:50 and polyclonal antibody NCL-CM I diluted 1:500 (Novocastra). Antigen retrieval was performed by microwave heating in citrate buffer, pH 6.0 (Cattoretti et al, 1992). A gastric carcinoma known to express p53 protein was used as positive control, and thyroglobulin and ret-40 were used as negative controls for the polyclonal and monoclonal antibodies respectively. Eighteen patients had two or three different blocks of biopsy tissues and 34 patients each had only one block of biopsy tissues available for immunohistochemical staining. Consecutive areas of the sections were examined independently by two observers who were blind to the clinical details of the patients. Overexpression was recorded when positive staining was present in more than 10% of the cells. The presence or absence of overexpression was, in all instances, distinct in that more than half of the cells in tumours overexpressing p53 stained positively and virtually no cells in the p53-negative cases demonstrated immunostaining. No borderline cases were identified.

The clinical records of the patients were retrieved from the database. All the patients had full staging studies consisting of routine haematological and serum biochemical parameters, whole body bone scintigraphy, and computerized tomography (CT) scans of the chest and local tumour. More recently, magnetic resonance imaging scans have replaced CT scans of the tumour site. All the patients received combination chemotherapy consisting of vincristine, doxorubicin, ifosfamide, cyclophosphamide, etoposide and actinomycin D. Treatment of the local tumour was surgery and/or radiotherapy. Full follow-up data were available for all patients.

The disease-free survival and overall survival were analysed according to the Kaplan–Meier method (Kaplan and Meier, 1958) only in patients without metastases at diagnosis. Disease-free survival was defined from the time of diagnosis to either the date of review or recurrence at local or distant sites. The overall survival was defined from the time of diagnosis to the date of review or date of death. Treatment-related death was counted as an event. The analyses for disease-free and overall survival were confined only to those patients without metastases at the time of diagnosis. Statistical comparisons of survival curves were performed by the Mantel–Cox log-rank test (Mantel, 1966). Cox proportional hazards regression model (Cox, 1972) was used to study the joint influence of factors which might alter survival time. Fisher's exact test was used to evaluate the relationship between p53 and tumour stage at diagnosis and response to chemotherapy; relative risk (RR) and 95% confidence intervals (CI) were calculated. $P$ value < 0.05 was considered statistically significant.

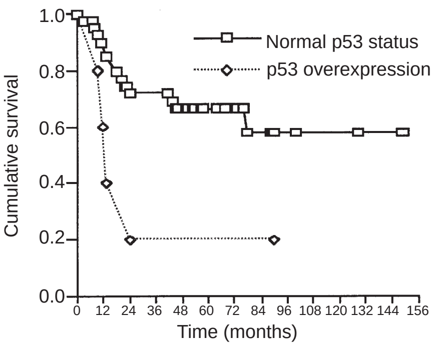

**Figure 1** Disease-free survival according to p53 protein status

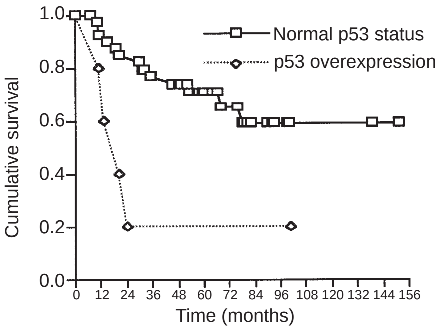

**Figure 2** Overall survival according to p53 protein status

### RESULTS

Fifty-two patients with Ewing's sarcoma of bone were studied. The mean age at diagnosis was 17 years (range 6–36 years). There were 30 males and 22 females. The tumours were located in bones of the extremities in 35 patients, pelvis in 13 patients, scapula in three patients and clavicle in one patient. This is similar to known distribution of Ewing's sarcoma (Huvos, 1991). Patients with pelvic, scapular and clavicular tumours were classified as having central tumours. Seven patients had metastases at diagnosis. The minimum follow-up was 30 months from the time of diagnosis.

p53 overexpression was demonstrated in seven patients (14%). There were consistent results in patients from whom multiple

*© Cancer Research Campaign 1999*

blocks of biopsy tissues were studied and between the two antibodies used. The relationship of p53 expression to the site of tumour, stage of tumour at diagnosis and the amount of necrosis within the tumour after preoperative chemotherapy were studied in all the patients. The disease-free survival and overall survival were studied only in those patients who did not have metastases at the time of diagnosis.

### p53 overexpression and site of tumours

There was no significant relationship between overexpression of p53 and location of tumours ($P > 0.9$). The site of the tumour was central in two patients and in the extremity bones in five patients, in those with p53 overexpression. In the patients with no p53 overexpression, the tumours were located in the central bones in 15 patients and in the extremity bones in 30 patients.

### Relationship between p53 overexpression, tumour stage at diagnosis and post-chemotherapy necrosis

Metastatic disease was present at diagnosis in two patients (29%) out of the seven patients in whom there was overexpression of p53 protein. Of the 45 patients who did not overexpress p53 protein, five patients (11%) had metastases at diagnosis.

The amount of necrosis within the excised tumour had been estimated from numerous samples taken from different parts of the excised tumours after a course of preoperative chemotherapy. Thirty-seven patients who did not have radiotherapy before excision of tumours were considered suitable for evaluation of the influence of overexpression of p53 protein on tumour necrosis. The amount of necrosis in the tumours was recorded as percentages of the whole tumour. In this study, patients were classified as 'good responders' when there was 90% or more necrosis and 'poor responders' when necrosis was less than 90%. Five patients (14%) out of the 37 patients studied demonstrated p53 overexpression. Of the five patients who expressed p53, only one patient (20%) had a good response to chemotherapy compared with 15 patients (47%) out of the 32 patients who did not express p53.

Although it appeared that there was a trend for Ewing's sarcoma overexpressing p53 protein to have more advanced disease at diagnosis and poorer response to chemotherapy, this observed difference did not reach statistical significance ($P = 0.4$, RR 2.6, 95% CI 1.2 – 5.9 for tumour stage; $P = 0.3$, RR 1.5, 95% CI 1.8–4.0 for necrosis).

### p53 over expression and patients' survival

Survival analysis was performed in 45 patients who did not have metastases at the time of diagnosis. These included five patients with overexpression of p53 protein, and all these had surgical excision and endoprosthetic reconstruction of tumours located in the extremity bones in four patients and pelvic bone in one patient. Overexpression of p53 protein was not demonstrated in 40 patients, 13 of these had central tumours and 27 had tumours of the extremity skeleton. The treatment of the local tumour in the latter group was surgical excision of expendable bones in ten patients, excision and endoprosthetic reconstruction in 21 and conventional radiotherapy in nine patients. The disease-free survival and overall survival of patients with p53 overexpression was significantly worse than those without p53 overexpression. The disease-free survival for patients with overexpression of p53 protein was 20% at 2 years and 5 years compared with 72% 2-year disease-free survival and 66% 5-year disease-free survival in the patients who did not overexpress p53 protein ($P = 0.02$), as shown in Figure 1.

The overall survival at 2 years and 5 years for patients with p53 overexpression was 20% compared with 85% 2-year survival and 71% 5-year survival for the patients who did not express p53 protein ($P = 0.01$), as shown in Figure 2.

The poorer overall survival for patients with expression of p53 protein was independent of tumour necrosis, site of tumour and the method of treatment of the local tumour (i.e. surgery and/or radiotherapy), with *P*-values of <0.05 according to the Cox proportional hazard model (Table 1). The poorer disease-free survival was also independent of site of tumour and method of treatment (Table 2).

**Table 1** Relative risk (RR) of death, confidence intervals (CI) and *P*-values estimated by Cox proportional hazard model analysis of the interaction between site of primary tumour, tumour necrosis, method of treatment of local disease and p53 overexpression

| | RR | CI | *P*-value |
|---|---|---|---|
| p53 | 3.6 | 1.9–18.4 | 0.01 |
| Method of local treatment | 1.2 | 0.72–2.1 | 0.38 |
| Necrosis | 1.8 | 0.90–2.5 | 0.05 |
| Site of tumour | 1.0 | 0.72–3.9 | 0.8 |

**Table 2** Relative risk (RR) of relapse of disease, confidence intervals (CI) and *P*-values estimated by Cox proportional hazard model analysis of the interaction between site of primary tumour, tumour necrosis, method of treatment of local disease and p53 overexpression

| | RR | CI | *P*-value |
|---|---|---|---|
| p53 | 2.8 | 1.17–16.6 | 0.02 |
| Method of local treatment | 1.1 | 0.93–1.8 | 0.30 |
| Necrosis | 1.5 | 0.83–2.7 | 0.07 |
| Site of tumour | 1.0 | 0.45–2.2 | 0.43 |

### DISCUSSION

Expression of p53 protein is a rare event in primary Ewing's sarcoma of bone. Only 14% of patients with Ewing's sarcoma of bone in this study demonstrated abnormalities of p53 protein expression. This figure is similar to those obtained by other authors (Kovar et al, 1993; Mangham et al, 1995). We have deliberately studied pretreatment biopsy tissues because the effect of chemotherapy or radiotherapy on p53 detection is uncertain. In those patients from whom multiple specimens of biopsy tissues were studied, the results in all the specimens were similar for each patient, demonstrating that the p53 protein expression is diffuse within the tumour.

There was a trend for patients with Ewing's sarcoma who expressed p53 protein to have more advanced stage disease at

© Cancer Research Campaign 1999

diagnosis and poorer response to chemotherapy than with patients who showed no expression of p53 protein and in whom the *p53* gene was presumably normal. Of the patient who demonstrated p53 expression, 29% had disseminated metastases at diagnosis compared with 11% of those without p53 expression. Only 20% of the patients with expression of p53 protein had a good response to chemotherapy, whereas 47% of patients who did not express p53 protein had a good response to preoperative chemotherapy. However, these differences were not statistically significant for the small number of patients in this study.

The differences in the disease-free survival and overall survival of patients with manifest expression of p53 protein and those without are particularly striking. Only 20% of patients who expressed p53 protein remained free of disease at 5 years compared with 66% of patients who did not express p53 protein. The overall survival of patients who expressed p53 protein was 20% at 2 years and 5 years compared with 85% and 71% at 2 years and 5 years, respectively, in those without expression of p53 protein. These differences are statistically significant, and are independent of location of the tumour, post-chemotherapy necrosis and type of treatment used for the local tumour.

Our results are similar to those obtained by Mangham et al (1995), who in a study of 38 patients with Ewing's sarcoma treated at the Massachusetts General Hospital, Boston, USA, found three patients (8%) to express p53 protein. All the patients who expressed p53 protein died by 15 months after diagnosis compared with 50% 5-year survival for patients who did not express p53 protein. This is the only other study of the prognostic relevance of p53 protein expression in Ewing's sarcoma.

The rarity of abnormalities of the *p53* gene and its protein in Ewing's sarcoma of bone suggests that this gene probably plays no significant role in the primary pathogenesis of Ewing's sarcoma of bone. However, it appears that abnormalities of the *p53* gene resulting in expression of the p53 protein occur later in the progression of Ewing's sarcoma, and may indicate the assumption of a more aggressive course.

At the present time, there is no histological grading system to indicate the biological behaviour of Ewing's sarcoma in different patients. There is an urgent need for identification of biological factors which may allow stratification of patients with localized Ewing's sarcoma of bone according to biological aggression of the tumour before commencement of treatment or early in the course of treatment. The present clinical prognostic factors only allow retrospective identification of those patients with poor prognosis during treatment. Routine examination of pretreatment biopsy tissues of Ewing's sarcoma for expression of p53 protein may allow identification of those tumours that are likely to have a more aggressive course resulting in poor prognosis on the current treatment regimen. This will be very useful in patients who present with localized Ewing's sarcoma, in whom there is presently no definite indicator of prognosis before commencement of treatment.

### CONCLUSION

Expression of p53 protein due to mutations of the *p53* gene is rare in patients with Ewing's sarcoma of bone. However, patients with Ewing's sarcoma who express p53 protein appear to present with more advanced disease and have poorer response to chemotherapy than patients who do not have expression of the p53 protein. The disease-free survival and overall survival of patients with Ewing's sarcoma with expression of p53 protein are significantly worse than those without p53 protein expression. Although the number of patients in this series is small, the data presented provide strong evidence that immunodetectable p53 expression is associated with poor prognosis in patients with Ewing's sarcoma of bone which needs to be investigated with a larger number of patients.

### REFERENCES

Baas IO, Mulder JR, Offerhaus GJA, Volgelstein B and Hamilton SR (1994) An evaluation of six antibodies for immunohistochemistry of mutant p53 gene product in archival colorectal neoplasms. *J Pathol* **172**: 5–12

Bartek J, Iggo R, Gannon J and Lane DP (1990) Genetic and immunocytochemical analysis of mutant p53 in human breast cancer cell lines. *Oncogene* **5**: 893–899

Cangir A, Vietti TJ, Gehan EA, Burgert EO, Thomas P, Tefft M, Nesbit ME, Kissane J and Pritchard D (1990) Ewing's sarcoma metastatic at diagnosis. *Cancer* **66**: 887–893

Cattoretti G, Becker MGH, Key G, Duchrow M, Schluter C, Galle J and Gerdes J (1992) Monoclonal antibodies against recombinant parts of ki-67 antigen (M1B1 and M1B3) detect proliferating cells in microwave processed fixed paraffin section. *J Pathol* **168**: 357–363

Cordon-Cardo C, Latres E, Drobnjak M, Oliva MR, Pollack D, Woodruff JM, Marechal V, Chen J, Brennan MF and Levine AJ (1994) Molecular abnormalities of mdm2 and p53 genes in adult soft tissue sarcomas. *Cancer Res* **54**: 794–799

Cox DR (1972) Regression models and life-tables (with discussion). *J R Stat Soc B* **34**: 187–220

Davidoff AM, Humphery PA, Iglehart JD and Marks JR (1991) Genetic basis for p53 overexpression in human breast cancer. *Proc Natl Acad Sci USA* **88**: 5006–5010

Esrig D, Spruck III CH, Nichols PW, Chaiwun B, Steven K, Groshen S, Chen S, Skinner DG, Jones PA and Cote RJ (1993) p53 nuclear protein accumulation correlates with mutation in the p53 gene, tumour grade and stage in bladder cancer. *Am J Pathol* **143**: 1389–1397

Ewing J (1921) Diffuse endothelioma of bone. *Proc N Y Pathol Soc* **21**: 17–24

Glass AG and Fraumeni JF (1970) Epidemiology of bone cancer in children. *J Natl Cancer Inst* **44**: 187–199

Greenblatt MS, Bennett WP, Hollstein M and Harris CC (1994) Mutations in the p53 tumour suppressor gene: clues to cancer aetiology and molecular pathogenesis. *Cancer Res* **54**: 4855–4878

Hsu H, Tseng H, Lai P, Lee P and Peng S (1993) Expression of p53 gene in 184 unifocal hepatocellular carcinomas: association with tumour growth and invasiveness. *Cancer Res* **53**: 4691–4694

Huvos AG (1991) Ewing's sarcoma. In *Bone Tumours*: *Diagnosis, Treatment and Prognosis*, 2nd edn. Mitchell L (ed.), pp. 523–552. W B Saunders: Philadephia

Isola J, Visakorpi T, Holli K and Kallioniemi O (1992) Association of overexpression of tumour suppressor protein p53 with rapid cell proliferation and poor prognosis in node-negative breast cancer patients. *J Natl Cancer Inst* **84**: 1109–1114

Kaplan FL and Meier P (1958) Non-parametric estimation from incomplete observations. *J Am Stat Assoc* **53**: 457–481

Kovar H, Auinger A, Jug G, Aryee D, Zoubek A, Salzer-Kuntschik M and Gadner H (1993) Narrow spectrum of infrequent p53 mutations and absence of *MDM* 2 amplification in Ewing's tumours. *Oncogene* **8**: 2683–2690

Lane DP (1993) A death in the life of p53. *Nature* **362**: 786–787

Larsson SE and Lorentzon R (1974) The geographic variation of the incidence of primary bone tumours in Sweden. *J Bone Joint Surg* **56A**: 592–600

Levine AJ (1992) The p53 tumour-suppressor gene. *N Engl J Med* **326**: 1350–1351

Mangham DC, Cannon A, Li X Q, Komiya S, Gebhardt MC, Springfield DS, Rosenberg AE and Mankin HJ (1995) p53 overexpression in Ewing's sarcoma/primitive neuroectodermal tumours is an uncommon event. *J Clin Pathol Mol Pathol* **48**: M79–M82

Mantel N (1966) Evaluation of survival data and two new rank order statistics arising in its consideration. *Cancer Chemother Rep* **50**: 163–170

Mendenhall CM, Marcus RB, Enneking WF, Springfield DS, Thar TL and Million RR (1983) The prognostic significance of soft-tissue extension in Ewing's sarcoma. *Cancer* **51**: 913–917

© Cancer Research Campaign 1999

Price CHG and GM Jeffree (1977) Incidence of bone sarcoma in SW England, 1946–74, in relation to age, sex, tumour site and histology. *Br J Cancer* **36**: 511–522

Sauer R, Jurgens H, Burgers JM, Dunst J, Hawlicek R and Michaelis J (1987) Prognostic factors in the treatment of Ewing's sarcoma. The Ewing's Sarcoma Study Group of the German Society of Paediatric Oncology CESS 81. *Radiat Oncol* **10**: 101–110

Toguchida J, Yamaguchi T, Wadayama BI, Kotoura Y, Yandell DW and Yamamuro T (1993) p53 tumour suppressor gene as the frequent target for somatic and germ-line mutation in bone and soft tissue sarcoma. In *Limb Salvage – Current Trends. Proceedings of the 7th International Symposium ISOLS, Singapore*. Tan SK (ed.)

© Cancer Research Campaign 1999

## References

[PDF_00355] Bartek J., Iggo R., Gannon J., Lane D. P. (1990). Genetic and immunochemical analysis of mutant p53 in human breast cancer cell lines.. Oncogene.

[PDF_00358] Cangir A., Vietti T. J., Gehan E. A., Burgert E. O., Thomas P., Tefft M., Nesbit M. E., Kissane J., Pritchard D. (1990). Ewing's sarcoma metastatic at diagnosis. Results and comparisons of two intergroup Ewing's sarcoma studies.. Cancer.

[PDF_00361] Cattoretti G., Becker M. H., Key G., Duchrow M., Schlüter C., Galle J., Gerdes J. (1992). Monoclonal antibodies against recombinant parts of the Ki-67 antigen (MIB 1 and MIB 3) detect proliferating cells in microwave-processed formalin-fixed paraffin sections.. J Pathol.

[PDF_00365] Cordon-Cardo C., Latres E., Drobnjak M., Oliva M. R., Pollack D., Woodruff J. M., Marechal V., Chen J., Brennan M. F., Levine A. J. (1994). Molecular abnormalities of mdm2 and p53 genes in adult soft tissue sarcomas.. Cancer Res.

[PDF_00371] Davidoff A. M., Humphrey P. A., Iglehart J. D., Marks J. R. (1991). Genetic basis for p53 overexpression in human breast cancer.. Proc Natl Acad Sci U S A.

[PDF_00379] Glass A. G., Fraumeni J. F. (1970). Epidemiology of bone cancer in children.. J Natl Cancer Inst.

[PDF_00381] Greenblatt M. S., Bennett W. P., Hollstein M., Harris C. C. (1994). Mutations in the p53 tumor suppressor gene: clues to cancer etiology and molecular pathogenesis.. Cancer Res.

[PDF_00384] Hsu H. C., Tseng H. J., Lai P. L., Lee P. H., Peng S. Y. (1993). Expression of p53 gene in 184 unifocal hepatocellular carcinomas: association with tumor growth and invasiveness.. Cancer Res.

[PDF_00389] Isola J., Visakorpi T., Holli K., Kallioniemi O. P. (1992). Association of overexpression of tumor suppressor protein p53 with rapid cell proliferation and poor prognosis in node-negative breast cancer patients.. J Natl Cancer Inst.

[PDF_00396] Kovar H., Auinger A., Jug G., Aryee D., Zoubek A., Salzer-Kuntschik M., Gadner H. (1993). Narrow spectrum of infrequent p53 mutations and absence of MDM2 amplification in Ewing tumours.. Oncogene.

[PDF_00399] Lane D. P. (1993). Cancer. A death in the life of p53.. Nature.

[PDF_00400] Larsson S. E., Lorentzon R. (1974). The geographic variation of the incidence of malignant primary bone tumors in Sweden.. J Bone Joint Surg Am.

[PDF_00402] Levine A. J. (1992). The p53 tumor-suppressor gene.. N Engl J Med.

[PDF_00408] Mantel N. (1966). Evaluation of survival data and two new rank order statistics arising in its consideration.. Cancer Chemother Rep.

[PDF_00410] Mendenhall C. M., Marcus R. B., Enneking W. F., Springfield D. S., Thar T. L., Million R. R. (1983). The prognostic significance of soft tissue extension in Ewing's sarcoma.. Cancer.

